# Molecular and Serological Footprints of *Mycobacterium avium* Subspecies Infections in Zoo Animals

**DOI:** 10.3390/vetsci7030117

**Published:** 2020-08-23

**Authors:** Marco Roller, Sören Hansen, Susanne Böhlken-Fascher, Tobias Knauf-Witzens, Claus-Peter Czerny, Ralph Goethe, Ahmed Abd El Wahed

**Affiliations:** 1 Wilhelma Zoological-Botanical Gardens Stuttgart, Wilhelma 13, D-70376 Stuttgart, Germany; marco.roller@tiho-hannover.de (M.R.); Tobias.Knauf-Witzens@wilhelma.de (T.K.-W.); 2 Department of Animal Sciences, Division of Microbiology and Animal Hygiene, Faculty of Agricultural Science, Georg-August-University, Burckhardtweg 2, D-37077 Göttingen, Germany; hansensoer@gmail.com (S.H.); susanne.boehlken-fascher@agr.uni-goettingen.de (S.B.-F.); cczerny@gwdg.de (C.-P.C.); 3 Institute for Microbiology, University of Veterinary Medicine Hannover, Foundation, Bischofsholer Damm 15, D-30173 Hannover, Germany; Ralph.Goethe@tiho-hannover.de; 4 Institute of Animal Hygiene and Veterinary Public Health, University of Leipzig, An den Tierkliniken 43, D-04103 Leipzig, Germany

**Keywords:** *Mycobacterium avium* subspecies *paratuberculosis*, Johne’s disease, paratuberculosis, *Mycobacterium avium* complex, zoo animals, serological assays, molecular assays, surveillance, monitoring

## Abstract

Background: Mycobacteria of the *Mycobacterium avium* complex (MAC) pose a significant risk to zoological collections. *Mycobacterium avium* subspecies *paratuberculosis* (MAP) is a member of MAC and the causative agent of Johne’s disease. Despite many reports in animals kept in zoological gardens, systemic surveillance has rarely been reported. Methods: In this study, archived serum samples collected from animal species at the Wilhelma Zoological and Botanical Gardens in Stuttgart, Germany, were screened for the presence of antibodies against MAC and MAP. In addition, molecular investigations were performed on necropsy, fecal, and environmental samples. Results: In total, 30/381 serum samples of various mammalian species were positive for MAC antibodies in ELISA, while one sample of a reticulated giraffe (*Giraffa camelopardalis reticulata*) was positive in MAP-specific ELISA. Samples from many species were positive in pan-*Mycobacterium* real-time PCR (40/43 fecal samples, 27/43 environmental samples, and 31/90 necropsy samples). Surprisingly, no sample was positive in the MAP-specific molecular assays. However, two environmental samples from primate enclosures were positive in *Mycobacterium avium* subspecies *hominissuis* (MAH)-specific real-time PCR. Conclusions: The results reveal serological indications of MAC infections in the zoological collection. However, the presence of a MAP-contaminated environment by a high-shedding individual animal or MAP-infected population is unlikely.

## 1. Introduction

The *Mycobacterium avium* complex (MAC) comprises *Mycobacterium avium* subspecies *avium* (MAA), *Mycobacterium avium* subspecies *paratuberculosis* (MAP), *Mycobacterium avium* subspecies *silvaticum* (MAS), and *Mycobacterium avium* subspecies *hominissuis* (MAH) [1,2,3]. Members of MAC are not species-specific and are frequently associated with animal or human diseases causing tuberculous lesions in lymph nodes and, occasionally, parenchymatous organs [4]. MAC mycobacteria and, in particular, MAP, pose a significant risk to zoological collections [5,6,7]. MAP is an acid-fast bacterium causing a contagious, chronic, and typically fatal, enteric disease of domestic and nondomestic ruminants named paratuberculosis (Johne’s disease (JD)) [8]. Common characteristics of paratuberculosis are latent and asymptomatic infections, while only a few animals of the herd develop clinical symptoms. Affected animals in the advanced stages of the disease suffer from wasting and gradual emaciation [9]. Clinical paratuberculosis has been diagnosed in a wide diversity of captive and free-ranging ruminant and pseudoruminant species, with considerably different clinical and pathological pictures [10,11]. However, MAP can also infect nonruminant animal species, with less clear symptoms [12].

In zoological gardens, various populations of diverse animal species are artificially managed in limited space. They are susceptible to epidemiological situations, similar to livestock herds, such as high animal density and exposure to a high concentration of infectious agents in the population.

Hence, MAC infection in zoo animals may be of significant relevance in terms of animal welfare and conservation efforts. Indeed, paratuberculosis outbreaks and systematic surveys for infection and disease have been reported for several zoos [13,14,15,16,17,18,19]. Some of these studies have included investigations in nonruminant species [20,21,22,23,24]. As a result, paratuberculosis has become an essential part of disease prevention and surveillance protocols of many zoological institutions, which include preshipment veterinary test requirements and strict hygiene and quarantine measures (see the workshop on diagnosis, prevention, and control of Johne’s disease in nondomestic hoofstock [25]).

**Figure 1:**
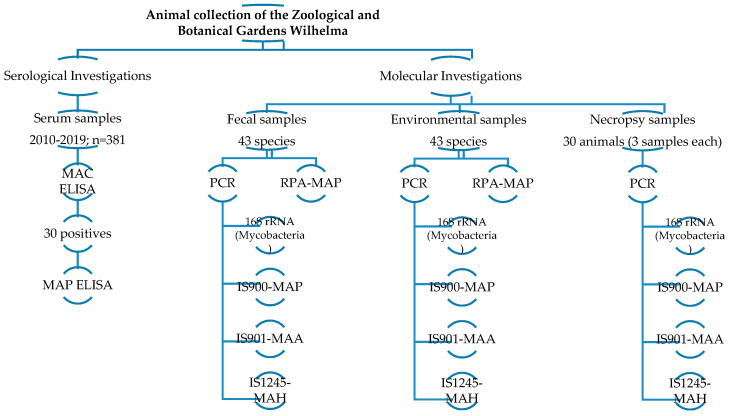
Flow chart of the methods used to screen the collected samples.

In this study, we performed a retrospective survey on frozen-stored mammalian serum samples collected in the Wilhelma Zoological and Botanical Gardens in Stuttgart, Germany, between 2010 and 2019. The screening was performed using an indirect ELISA specific for the antibody of MAC, as well as a second assay targeting the MAP-specific antibody. In order to identify active or subclinical MAP infection, a mobile molecular assay based on a recombinase polymerase amplification (RPA) assay was applied in the zoological garden for onsite molecular detection of MAP in fecal and environmental samples. The results of the field settings were followed by laboratory-based real-time PCR targeting pan-*Mycobacterium* species MAP, MAA, and MAH (Figure 1).

## 2. Materials and Methods

### 2.1. Sample Collection

Ethical statement: All samples were collected in accordance with the guidelines of good veterinary practice and the law of the Federal Republic of Germany. The regional administrative authority in Stuttgart, Germany (Regierungspräsidium Stuttgart, Abteilung 3-Landwirtschaft, Ländlicher Raum, Veterinär- und Lebensmittelwesen), has been notified of the study under file number 35-9185.82/0347.

#### 2.1.1. Serum Samples

A serologic survey for *Mycobacterium avium* complex antibodies was performed on frozen-stored serum samples from mammalian species, collected between 2010 and 2019. A total of 381 samples from 296 individuals, representing 62 different species in 22 families in 6 orders, were examined (Table 1). The indication for examination, age of the animal, sampling date, and medical history of each individual was saved in records in the zoo database.

#### 2.1.2. Fecal and Environmental Samples

Fecal samples and environmental samples were taken from 22 artiodactyl and 18 primate species, as well as from Rock hyraxes (*Procavia capensis*) and Malayan tapirs (*Tapirus indicus*) (Table 2). For a better risk assessment of MAP presence, pooled fecal samples of approximately 10–100 g from each species were collected for a period of seven consecutive days. To prevent or minimize contamination by MAP or other mycobacteria distributed in the environment, fresh fecal samples were carefully picked from the compound floors on a daily basis. Since sample collection took place during the daily cleaning routine, no animal was subjected to any additional impairment or stress during the sample collection.

To identify contaminated environments, environmental material from indoor and outdoor enclosures was collected by a one-time sampling with a pair of absorptive boot swabs (HELE GmbH, Heilsbronn, Germany). The sampling method described by Eisenberg et al. [26] for the collection of environmental samples from common locations in dairy herds for MAP detection was adjusted for collection in zoo enclosures. Sampling was conducted, especially in sectors with high animal traffic, by meandering expiration of the main paths of the animals, as well as bedding, feeding, and dunging areas. A minimum of 100 steps was walked, preferably in areas with high numbers of fecal residues and contaminations. Again, no animal was subjected to additional impairment or stress by these examinations. Both fecal and environmental samples were tested onsite in a mobile suitcase laboratory for the rapid detection of MAP in fresh samples. Subsequently, all samples were kept frozen at −20 °C for up to six months until further processed in a routine diagnostic laboratory.

#### 2.1.3. Tissue Samples

All deceased or euthanized zoo animals of interest were sampled during routine postmortem examinations at the pathology department of the Chemical and Veterinary Investigation Office in Stuttgart, Germany (CVUA-S), between 2017 and 2019. Tissue samples of the ileum, the ileocecal lymph node, and intestinal contents were collected from 15 primates, 13 artiodactyls, and 2 Rock hyraxes. All samples were stored at −20 °C until further investigations.

### 2.2. Serological Survey

#### 2.2.1. *Mycobacterium avium* Complex (MAC) ELISA

All serum samples were tested with a commercial indirect ELISA (ID Screen *Mycobacterium avium* Indirect Multi-species, ID.vet Innovative Diagnostics, Grabels, France) for immunoglobulin G (IgG) antibodies against *Mycobacterium avium* complex (anti-multispecies IgG-HRP conjugate-concentrated, 10×) according to the manufacturer’s instructions. Each sample was tested individually. The negative and positive controls, provided by the manufacturer, were run in triplicate. Plates were analyzed using an automated ELISA plate reader (SunriseTM, Tecan Trading AG, Switzerland). Results were calculated as the mean sample-to-positive ratio (S/P ratio) = (OD450 of sample − OD450 of negative control)/(OD450 of positive control − OD450 of negative control). According to the manufacturer’s instructions, readings equal to or below 40% of the positive control serum OD (Optical density) were considered as negative, readings equal to or greater than 50% were considered as positive, and readings between 40% and 50% were scored as doubtful. Doubtful and positive results were tested again in duplicate with the same protocol.

#### 2.2.2. *Mycobacterium avium* Subspecies Paratuberculosis (MAP) ELISA

Positive and suspicious serum samples in the ID Screen ELISA (n = 30) were subsequently analyzed for the presence of antibodies against MAP using a commercial indirect ELISA (IDEXX Paratuberculosis Screening Ab Test, IDEXX Laboratories Inc., Westbrook, Maine, USA), following the instructions of the manufacturer. The detection of bound antibodies was based on a horseradish peroxidase (HRPO) protein-G conjugate, which reacts with antibodies of multiple animal species. Positive ELISA results were compared with the medical history of each individual, containing recorded clinical symptoms as well as antemortem diagnostics and routine measurements. If available, the cause of death and postmortem reports were also examined for possible connections.

### 2.3. Molecular Survey

For the detection of MAP DNA at the zoo premises, a mobile suitcase laboratory operating an isothermal amplification technique (recombinase polymerase amplification (RPA)) was used to test the fecal and environmental samples immediately after sample collection. Considering that MAP form clusters in different sample matrices [27], DNA was extracted from several portions of each pooled sample per animal species and environmental boot swab to compensate for nonhomogeneous bacterial distribution patterns. For RPA, 5 portions (~100 µg) of each fecal sample and 3 portions (~1 × 1 cm) of each boot swab were extracted using SpeedXtract (QIAgen, Hilden, Germany) and RPA, as previously described [28,29]. Thereafter, fecal and environmental samples were stored at −20 °C until shipped for laboratory screening with three real-time PCR assays targeting pan-*Mycobacterium*, MAP, MAA, and MAH.

The pan-*Mycobacterium* assay is based on the amplification of the 16S rRNA gene from *Mycobacterium* species, which amplifies the hypervariable region A [30]. For the detection of MAP DNA, a real-time PCR assay based on IS900 was used [31]. The multiplex real-time PCR assay based on the simultaneous detection of specific insertion sequences, IS901 and IS1245, was used for the detection of MAA and MAH, respectively [32]. In total, 2 portions (~50–120 µg) of each fecal sample and 2 parts (~1 × 1 cm) of each boot swab were extracted, as previously described [33,34]. DNA extracts were examined individually for IS900 detection or pooled for detection of 16S rRNA, IS901, and IS1245.

Amplification and fluorescence detection were performed on the Light Cycler 480 System using 96-well PCR plates (Roche Molecular Diagnostic, Mannheim, Germany). A total reaction volume of 20 μL per well was used, containing 10 μL of Light Cycler 480 Probes Master mix, 0.5 μL of each upstream and downstream primer (10 pmol/μL), 1 μL of the probe (10 pmol/μL) [30,31,32], as well as 3 μL of molecular biology water, and 5 μL of the extracted DNA template. The amplification process started with an initial preincubation step at 95 °C for 10 min, followed by 40 cycles at 95 °C for 15 s, 60 °C for 30 s, and 72 °C for 35 s, followed by a final cooling step at 40 °C for 30 s. Reference strains (MAP: ATCC 19698; MAA: DSM44156; MAH: ATCC 700898) were used as positive controls, whereas sterile distilled water was used as a negative control.

## 3. Results

### 3.1. Serological Survey

**Figure 2:**
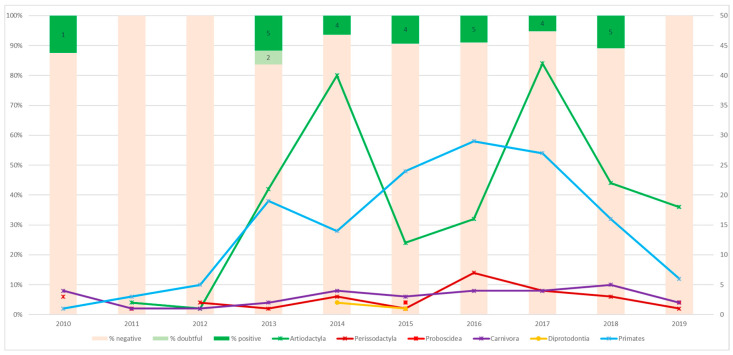
Results of the *Mycobacterium avium* complex antibody ELISA (column). Percentages of negative, positive, and doubtful samples per year were indicated. The colored lines indicate the number of samples tested from the different taxonomic orders. Numbers in green boxes indicate the positive or doubtful samples per year; 2010: Sumatran orangutan; 2013: alpaca, 2 Persian fallow deer, and 2 doubtful samples, namely, Eastern bongo, Mishmi takin; 2014: onager, collared peccary, lowland anoa, bonobo; 2015: western lowland gorilla, Buru babirusa, onager, black howler; 2016: brown spider monkey, western lowland gorilla, onager, Gelada baboon; 2017: domestic cattle, black howler, Barbary sheep, Persian fallow deer; 2018: Eastern Javan langur, lesser kudu, vicuna, reticulated giraffe, Buru babirusa.

Among the 381 serum samples, 28 were positive (24 individuals) and 2 were suspicious in the MAC indirect multispecies ELISA (Table 1). Eight nonhuman primates in seven species harbored MAC antibodies, including one Sumatran orangutan (*Pongo abelii*), one bonobo (*Pan paniscus*), and two Western lowland gorillas (*Gorilla gorilla gorilla*). Antibodies could also be detected in two equids (one species), two suids (two species), two camelids (two species), five cervids (one species; 3 positive and two suspicious results), six bovids (six species), and one reticulated giraffe. Not all animals were positive in the same year, but as indicated in Figure 2, around 4–5 animals tested positive per year, starting in 2013.

The screening of all positive and suspicious samples in the MAP antibody-specific ELISA revealed only one positive result in the reticulated giraffe. All other samples gave negative results.

### 3.2. Molecular Survey

All fecal (n = 43) and environmental (n = 43) samples were negative in the MAP/RPA assay directly performed in the zoological garden. Negative results were also obtained in these samples when tested with MAP/real-time PCR. Pan-*Mycobacterium*-16S rRNA was detected in 40 of 43 fecal samples and in 27 of 43 samples from the enclosures (Table 2). All artiodactyl and primate samples were negative in the MAA–IS901–real-time PCR. Two environmental samples from the enclosures of the Eastern Javan langurs (*Trachypithecus auratus auratus*) and one of two family groups of bonobos were positive for MAH–IS1245–real-time PCR. It is worth mentioning that at the time of sampling, no clinical case suggestive for paratuberculosis or any other mycobacterial disease was evident at the zoo. Fecal consistency was representative of healthy individuals of the respective species. The population size of the animal species at the time of sampling is given in Table 2.

Samples of the ileum, the ileocecal lymph node, and intestinal contents collected during routine postmortem examinations of deceased or euthanized zoo animals originated from 15 primates, 13 artiodactyls, and 2 Rock hyraxes. Positive results were only obtained in the pan-*Mycobacterium*–16S rRNA real-time PCR (Table 3).

## 4. Discussion

Several publications and review articles have addressed the importance of prevention and control of paratuberculosis in zoological gardens, where the disease can threaten the animal collection of exotic and often endangered species [13,14,15,16,20,35,36]. Nevertheless, systematic surveys of MAP infection are scarce and focus mostly on various ruminant species. The scope of the present study was to investigate the presence of MAC and MAP at the Wilhelma Zoological and Botanical Gardens, Stuttgart, Germany, applying serological and molecular examination techniques.

Frozen-stored serum samples from various mammalian species were screened with an indirect multispecies ELISA for the presence of antibodies against MAC. The retrospective serologic survey revealed several positive-tested individuals over the years. Our results suggest seroprevalence of infection in various mammals within the animal collection, acknowledging that samples for examination were selected at random. The same ELISA has been used before in a retrospective serologic survey by Matos et al. in free-ranging wild mammals in Portugal [37]. They showed evidence for MAC antibodies in Canidae, Mustelidae, and Suidae families, which confirms that MAC can infect animals of multiple taxonomic groups. Based on a calculated sensitivity between 34.5% and 44% and a specificity of 100%, an actual higher prevalence was assumed [37]. This could also apply to this study, acknowledging that the test has not yet been established or reported in a zoological collection. In our study, positive results were obtained from nonhuman primates, Equidae, Suidae, Camelidae, Cervidae, and Bovidae. However, in contrast to the study from Portugal, no positive results were found in carnivores (Canidae, Felidae, Otariidae, Procyonidae, and Ursidae), which might be explained by the lower sample number of this study. The comparison with the medical histories and postmortem reports of MAC antibody-positive animals (available for 13 of 26 individuals) revealed a possible explanation for the positive result in two cases: one female onager, euthanized because of laminitis and tendon rupture, showed a lump in the abdominal wall with questionable Ziehl–Neelson staining. However, MAA, MAH, and *Mycobacterium tuberculosis* complex (MTC) real-time PCR, as well as MTC culture, were negative in investigations at CVUA-S. Therefore, atypical mycobacteria were considered likely. Clinical symptoms matched in an aged eastern bongo, euthanatized because of chronic diarrhea and severe emaciation. Postmortem examination revealed catarrhal enteritis, but MAP could not be detected by real-time PCR. Interestingly, *Mycobacterium avium* DNA was detected by real-time PCR in a fecal sample of another bongo a few years earlier, but again, MAP was excluded by real-time PCR and culture. In all other ELISA-positive cases, the medical history could not be related to the positive test results.

MAC antibody-positive samples were subsequently tested in a MAP-specific ELISA. This test has previously been applied for antibody detection in frozen-stored serum samples of a zoological institution, where positive results were detected in several exotic ruminants and Malayan tapirs [13]. The analysis of all positive and suspicious samples for MAC antibodies in this study revealed only one positive result in a serum sample from a female reticulated giraffe, drawn half a year prior to its death. An additional serum sample from the day before death also tested positive for MAP. The animal died of a scirrhous hepatocellular adenocarcinoma and a scirrhous bile adenocarcinoma, with metastases in the lungs. Indications for MAP infection or clinical paratuberculosis could not be demonstrated during postmortem and follow-up examination. To our knowledge, the presence of MAP in giraffes has only been reported in two studies. Stevenson et al. [38] mentioned a positive result from a captive giraffe, which was analyzed by restriction fragment length polymorphism (RFLP). Ball et al. [39] reported the intensive screening of a reticulated giraffe that was kept in isolation after a single positive fecal culture.

No MAP-positive results were detected by both RPA and real-time PCR. Since MAP is shed in clusters and only a low DNA copy number of MAP was expected in tested samples, a total of five portions of each pooled fecal sample and three parts of each boot swab were used for molecular assays. Accordingly, based on a sample-collecting strategy and the findings, we conclude that during the time of examination, the presence of a high-shedding animal or a MAP-infected population in the collection must be considered unlikely.

Overall, these results are very valuable for the health monitoring and management of the zoological collection, since this is the first extensive MAP screening at this institution. Nevertheless, negative test results do not exclude the presence of MAP, and positive results must be interpreted with caution. Culture- or PCR-positive fecal samples provide very strong evidence of exposure but do not confirm infection as a fecal “pass-through” phenomenon, and therefore, a passive shedding without infection may occur subsequent to oral ingestion of MAP [40]. Additionally, intermittent excretion of the pathogen and shedding of MAP in clusters and nests may lead to negative results in shedding-free intervals [31]. The prolonged subclinical incubation period makes continuous and repeated disease surveillance advisable. The detection of low-level shedders must include the collection and evaluation of different sample matrices at different times and should always include the medical history of individuals and herds. Positive diagnostic results in several different samples increase the confidence about infection, which is important for disease management.

Likewise, a MAP-contaminated environment may be considered unlikely in the present zoo due to the results of intensive sampling of the surroundings of the animals. The pathogen can persist and survive in the environment for more than one year [41]. Negative results can be explained by the regular cleaning of indoor and outdoor enclosures and the daily removal of feces, leading to a pathogen-poor environment and reduced infection pressure at the zoo.

The setup of a mobile suitcase laboratory allows the rapid detection of MAP shedders directly onsite to make a highly specific and sensitive diagnosis [29]. The quick and uncomplicated setup can be easily integrated into existing monitoring protocols and may also provide diagnostic options for other important pathogens in zoological institutions. RPA has been adopted for other emerging and neglected infectious diseases [42,43,44,45,46]. Negative RPA results in our study were confirmed by negative results in real-time IS900 PCR.

Bacteriologic cultural investigations were not attempted in this screening study due to long cultivation times. However, cultivation is advisable in justified suspected cases in individual animals.

Positive results in the pan-mycobacterial 16S rRNA real-time PCR assay could be explained with the presence of environmental and atypical mycobacterial species. Nontuberculous mycobacteria (NTM) have a ubiquitous distribution and can be found in the environment of humans [47], as well as in the environment of animals in zoological gardens. In a recent study, pan-mycobacterial 16S rRNA was detected in 26.8% of rectal swabs and 2.5% of oral swabs from mammals (n = 860) and birds (n = 230) kept in six zoological gardens in Germany [48]. The prevalence in this study for the Wilhelma Zoological and Botanical Gardens was 25% for rectal swabs and 2.95% for oral swabs. Clinical symptoms were not observed in the tested animals. The higher number of positive results in our study could be explained by the applied sampling method, where feces were repeatedly collected noninvasively from the compounds for seven consecutive days. Mycobacteria of MTC were considered unlikely as the zoo has been officially free from tuberculosis for more than 25 years, and animals are screened on a regular basis in order to obtain tuberculosis-free status and to ensure a regulated transport of individual animals (Council Directive 92/65/EEC (Balai Directive)).

The multiplex real-time PCR for the simultaneous detection of MAA and MAH was positive for a MAH IS1245 insertion sequence in environmental samples of the enclosures of Eastern Javan langurs and one family group of bonobos. The detection of MAH has also been described in other species: cervids [49,50], cattle [51], horses [52], dogs [53,54,55,56], one cat [57], one blue-fronted Amazon parrot (*Amazona aestiva*) [58], and in naturally infected captive water birds [59]. In a zoological garden, MAH infection was diagnosed in bongo antelopes (*Tragelaphus eurycerus*). The animals suffered from emaciation, and postmortem examination revealed acid-fast bacteria and nodular lesions in the lungs of the examined animals. Environmental examination by bacteriologic culture revealed MAH in mulch bark, peat, and soil [60]. *Mycobacterium avium* is commonly isolated from captive elephants and is not generally associated with disease, although a single fatal case of mycobacterial lung infection due to MAH has been reported in an African elephant (*Loxodonta africana*) [61]. A coinfection of MAH and MAP has been reported by Glawischnig et al. [62] in free-ranging red deer (*Cervus elaphus hippelaphus*), with signs of diarrhea, severe weight loss, and emaciation. Postmortem examination revealed lymphadenitis associated with grossly enlarged mesenteric lymph nodes, as well as multiple caseous or purulent nodular lesions in the thickened wall of the intestines. Although no clinical symptoms have been reported within nonhuman primates, the potential pathogenicity of MAH for these animals should be taken into account and needs to be ascertained. Infections and resulting disease caused by MTC and other mycobacteria have been reported for many primate species in zoological gardens [63].

MAA was not detected in our investigations, which can be explained by our focus on the selection of mammalian species. In addition to other mycobacteria, MAA is primarily a causative agent of avian mycobacteriosis [7], although infections in mammals have been described in zoological gardens (Bengal tiger (*Panthera tigris*) [64]; Asian elephant (*Elephas maximus*) [65]).

The characteristics of MAC and MAP infections, as well as the possibilities of their diagnosis and management, depend on the conditions and structure of the affected zoological institutions. The results of this study are, therefore, not representative of zoos in general. A number of tests are available for the surveillance and detection of zoo animals with paratuberculosis [11], but data on specificity and sensitivity of the applied tests are missing or incomplete when screening exotic species. However, the methods applied here could provide a feasible opportunity to investigate the occurrence of infections in the susceptible animal population, especially in zoological institutions, where a higher prevalence is expected.

## 5. Conclusions

In conclusion, the present study reports the extensive surveillance for MAC and MAP exposure and infection in the animal collection of the Wilhelma Zoological and Botanical Gardens, Stuttgart, Germany.

Based on our results, further studies on the occurrence and epidemiology of MAP are required to prevent the spread and transmission in and between zoological gardens. The inclusion of other mycobacteria of MAC in these studies is an important component for species differentiation and a better understanding of their importance to captive wildlife. Further investigations should consider the use and implementation of point-of-need diagnostic systems for the rapid onsite detection of MAP and other pathogens in disease prevention and control measures.

## Figures and Tables

**Table 1:** Results of screening serum samples from different mammalian species using *Mycobacterium avium* complex indirect ELISA.

Order	Family	Species	Common Name	No. Tested	Ind. Tested	No. (+)	Ind. (+)
Diprotodontia	*Macropodidae*	*Macropus rufus*	Red kangaroo	3	3		
Proboscidea	*Elephantidae*	*Elephas maximus*	Asian elephant	5	3		
Primates	*Lemuridae*	*Varecia rubra*	Red ruffed lemur	1	1		
	*Atelidae*	*Ateles hybridus*	Brown spider monkey	16	10	2	1
		*Alouatta caraya*	Black howler	6	4	2	1
	*Pitheciidae*	*Pithecia pithecia*	White-faced saki	4	2		
	*Cercopithecidae*	*Trachypithecus auratus auratus*	Eastern Javan langur	11	7	1	1
		*Macaca fuscata*	Japanese macaque	9	8		
		*Mandrillus leucophaeus*	Drill	9	3		
		*Theropithecus gelada*	Gelada baboon	33	32	1	1
	*Hylobatidae*	*Hylobates lar*	Lar gibbon	7	5		
	*Pongidae*	*Pongo abelii*	Sumatran orangutan	9	6	1	1
		*Gorilla gorilla gorilla*	Western lowland gorilla	17	11	2	2
		*Pan paniscus*	Bonobo	22	12	1	1
Carnivora	*Procyonidae*	*Nasua nasua*	South American coati	1	1		
	*Ursidae*	*Ursus arctos syriacus*	Syrian brown bear	4	3		
		*Ursus maritimus*	Polar bear	1	1		
		*Tremarctos ornatus*	Spectacled bear	2	2		
	*Canidae*	*Speothos venaticus*	Bush dog	1	1		
		*Chrysocyon brachyurus*	Maned wolf	3	3		
	*Otariidae*	*Zalophus californianus*	California sea lion	3	3		
	*Felidae*	*Panthera uncia*	Snow leopard	5	4		
		*Panthera leo persica*	Asiatic lion	1	1		
		*Panthera onca*	Jaguar	1	1		
		*Panthera pardus saxicolor*	Persian leopard	5	3		
		*Panthera tigris sumatrae*	Sumatran tiger	2	2		
		*Acinonyx jubatus*	Cheetah	1	1		
Perissodactyla	*Rhinocerotidae*	*Rhinoceros unicornis*	Indian rhino	4	2		
	*Tapiridae*	*Tapirus indicus*	Malayan tapir	1	1		
	*Equidae*	*Equus asinus asinus*	Poitou-ass	2	1		
		*Equus africanus somaliensis*	Somali wild ass	4	3		
		*Equus ferus przewalskii*	Przewalski’s horse	2	2		
		*Equus grevyi*	Grevy’s zebra	8	6		
		*Equus hemionus onager*	Onager	3	2	3	2
Artiodactyla	*Suidae*	*Sus scrofa f. domestica*	Domestic pig	2	2		
		*Babyrousa babyrussa*	Buru babirusa	8	7	2	1
		*Pecari tajacu*	Collared peccary	1	1	1	1
	*Hippopotamidae*	*Hippopotamus amphibius*	Common hippopotamus	1	1		
		*Choeropsis liberiensis*	Pygmy hippopotamus	1	1		
	*Camelidae*	*Camelus bactrianus*	Bactrian camel	4	3		
		*Lama pacos*	Alpaca	16	11	1	1
		*Vicugna vicugna*	Vicuña	5	5	1	1
	*Cervidae*	*Dama mesopotamica*	Persian fallow deer	14	14	3 (2)	3 (2)
	*Giraffidae*	*Giraffa camelopardalis reticulata*	Reticulated giraffe	2	2	1	1
		*Okapia johnstoni*	Okapi	8	5		
	*Bovidae*	*Tragelaphus eurycerus*	Eastern bongo	5	5	1	1
		*Tragelaphus imberbis*	Lesser kudu	14	12	1	1
		*Bos taurus f. domesticus*	Domestic cattle	13	9	1	1
		*Bubalus depressicornis*	Lowland anoa	2	2	1	1
		*Bison bison*	American bison	3	3		
		*Addax nasomaculatus*	Addax	1	1		
		*Oryx dammah*	Scimitar-horned oryx	4	4		
		*Kobus ellipsiprymnus*	Waterbuck	1	1		
		*Gazella dorcas*	Dorcas gazelle	7	4		
		*Oreamnos americanus*	Rocky Mountain goat	4	2		
		*Budorcas taxicolor taxicolor*	Mishmi takin	5	3	1	1
		*Ovis aries f. domestica*	Domestic sheep	1	1		
		*Ammotragus lervia*	Barbary sheep	19	16	1	1
		*Capra falconeri*	Markhor	24	22		
		*Capra aegagrus*	Wild goat	5	5		
		*Capra hircus f. domestica*	Dwarf goat	1	1		
		*Capra ibex*	Alpine ibex	4	3		
**TOTAL**		**62**		**381**	**296**	**28 (2)**	**24 (2)**

No. = number of tests; Ind. = number of individuals; No. (+) = number of positive tests; Ind. (+) = number of positive individuals.

**Table 2:** Real-time PCR (MAC/16S rRNA, MAH/IS1245) results of collected fecal samples and environmental samples.

	Family	Scientific Name	Common Name	Pop.	Fecal Samples	Environmental Samples	
					16S rRNA	16S rRNA	IS 1245
PRIMATES	Callimiconidae	*Callimico goeldii*	Goeldi’s monkey	1.3	pos	pos	-
		*Leontopithecus chrysomelas*	Golden-headed lion tamarin	1.1	pos	pos	-
		*Saguinus imperator subgrisescens*	Bearded emperor tamarin	0.2	pos	pos	-
		*Cebuella pygmaea*	Pygmy marmoset	2.2.1	pos	pos	-
	Atelidae	*Ateles hybridus*	Brown spider monkey	1.2	-	pos	-
		*Alouatta caraya*	Black howler	2.2	pos	pos	-
	Cebidae	*Saimiri boliviensis boliviensis*	Bolivian squirrel monkey	4.5	pos	pos	-
	Pitheciidae	*Pithecia pithecia*	White-faced saki	1.3	pos	pos	-
	Cercopithecidae	*Mandrillus leucophaeus*	Drill	1.1	pos	pos	-
		*Theropithecus gelada*	Gelada baboon	8.28.4	pos	pos	-
		*Macaca fuscata*	Japanese macaque	2.3	pos	pos	-
		*Trachypithecus auratus auratus*	Eastern Javan langur	3.7	pos	pos	pos
	Hylobatidae	*Hylobates lar*	Lar gibbon	1.1	pos	-	-
	Lemuridae	*Varecia rubra*	Red ruffed lemur	0.1	pos	pos	-
	Lorisidae	*Nycticebus coucang*	Slow loris	0.1	pos	pos	-
	Pongidae	*Pan paniscus*	Bonobo (Group I)	2.4	pos	pos	-
		*Pan paniscus*	Bonobo (Group II)	4.6	pos	pos	pos
		*Gorilla gorilla gorilla*	Western lowland gorilla	5.6	pos	pos	-
		*Pongo abelii*	Sumatran orangutan	0.2	pos	pos	-
ARTIODACTYLA	Bovidae	*Capra hircus f. domestica*	Dwarf goat	1.6	pos	pos	-
		*Capra ibex*	Alpine ibex	1.6	pos	-	-
		*Bison bison*	American bison	1.2	pos	pos	-
		*Tragelaphus eurycerus*	Eastern bongo	2.4.1	pos	pos	-
		*Gazella dorcas*	Dorcas gazelle	2.5	pos	pos	-
		*Bos taurus f. domesticus*	Domestic cattle	0.2.2	-	-	-
		*Ovis aries f. domestica*	Domestic sheep	1.6	pos	pos	-
		*Tragelaphus imberbis*	Lesser kudu	0.9	pos	-	-
		*Bos taurus f. domesticus*	Domestic cattle	0.5.1	pos	-	-
		*Ammotragus lervia*	Barbary sheep	4.8.2	pos	-	-
		*Capra falconeri*	Markhor	1.7	pos	-	-
		*Budorcas taxicolor taxicolor*	Mishmi takin	1.1	pos	-	-
		*Oryx dammah*	Scimitar-horned oryx	0.2.2	pos	-	-
		*Oreamnos americanus*	Rocky Mountain goat	0.2	pos	-	-
		*Capra hircus f. domestica*	Black Forest goat	0.2	pos	-	-
		*Ovis aries f. domestica*	Domestic sheep	1.6	pos	-	-
		*Bison bison bonasus*	European Bison	1.0	pos	pos	-
	Camelidae	*Lama pacos*	Alpaca	0.14	pos	-	-
		*Camelus bactrianus*	Bactrian camel	0.3	pos	pos	-
		*Vicugna vicugna*	Vicuña	0.1	pos	-	-
	Cervidae	*Dama mesopotamica*	Persian fallow deer	0.4	pos	-	-
	Giraffidae	*Giraffa camelopardalis reticulata*	Reticulated giraffe	2.2	pos	pos	-
a	Procaviidae	*Procavia capensis*	Rock hyrax	0.3.5	-	-	-
b	Tapiridae	*Tapirus indicus*	Malayan tapir	2.0	pos	pos	-

a = Hyracoidea; b = Perissodactyla; Pop. = population (1.0—male, 0.1—female, 0.0.1—sex unknown); pos = positive; - = negative.

**Table 3:** MAC/16S rRNA real-time PCR results of tissue samples (ileum, ileocecal lymph node, and intestinal contents) of deceased or euthanized zoo animals.

Scientific Name	Common Name	Sex	Age (Year)	Cause of Death	Ileum	Ileocecal Lymph Node	Feces
*Leontopithecus chrysomelas*	Golden-headed lion tamarin	1.0	1	Deceased/Angiostrongylus			
		1.0	1	Euthanasia/Angiostrongylus	(pos)		
*Saguinus imperator subgrisescens*	Bearded emperor tamarin	1.0	10	Euthanasia/Phlegmon (Thigh)			
*Cebuella pygmaea*	Pygmy marmoset	1.0	0	Deceased/Lissencephaly		pos	
		0.1	0	Deceased/Trauma			
*Callithrix geoffroyi*	Geoffroy’s tufted ear marmoset	0.1	21	Deceased/Neoplasia (Uterus)			pos
*Ateles hybridus*	Brown spider monkey	1.0	1	Deceased/Trauma/Enteritis (para.)			(pos)
*Alouatta caraya*	Black howler	1.0	21	Euthanasia/Colitis, Nephritis (bact.)		(pos)	pos
*Theropithecus gelada*	Gelada baboon	0.1	24	Deceased/Age-related/Cardial Disease	pos		pos
		1.0	1	Euthanasia/Trauma			pos
		0.1	2	Euthanasia/Trauma			pos
		0.1	22	Deceased/Cardiomyopathy/Aneurysm			pos
*Trachypithecus auratus auratus*	Eastern Javan langur	0.1	21	Euthanasia/Abscess (Lung)/Age-related	pos	(pos)	pos
*Hylobates lar*	Lar gibbon	0.1	34	Deceased/Pleuropneumonia, Septicaemia (bact.)			pos
		1.0	43	Euthanasia/Septicaemia (bact.)			pos
*Ammotragus lervia*	Barbary sheep	0.1	7	Euthanasia/Trauma/Cachexia			
		1.0	0	Deceased/Premature Birth			
		0.1	4	Culling	(pos)		
*Ovis aries f. domestica*	Domestic sheep	1.0	9	Culling			pos
		0.1	7	Culling			pos
		0.1	8	Culling	pos		
		0.1	4	Culling			(pos)
		0.1	3	Culling			pos
		0.1	6	Culling	pos		
*Oryx dammah*	Scimitar-horned oryx	1.0	0	Euthanasia/Enteritis, Meningoencephalitis (bact.)			pos
*Budorcas taxicolor taxicolor*	Mishmi takin	1.0	21	Euthanasia/Age-related/Arthrosis	pos		pos
*Oreamnos americanus*	Rocky Mountain goat	0.1	14	Deceased/Septicaemia (bact.)		pos	pos
*Dama mesopotamica*	Persian fallow deer	0.1	11	Deceased/Age-related/Abomasitis (bact.)			pos
*Procavia capensis*	Rock hyrax	0.1	7	Deceased/Cachexia/Fatty Liver			pos
		0.1	8	Deceased/Cachexia/Acute Circulatory Collapse	(pos)		

Sex = 1.0—male and 0.1—female; age (y) = age in years at time of death; cause of death according to the necropsy report; pos = positive; (pos) = suspicious.

## References

[1] Thorel M.F., Krichevsky M., Lévy-Frébault V.V. (1990). Numerical taxonomy of mycobactin-dependent mycobacteria, emended description of *Mycobacterium avium*, and description of *Mycobacterium avium* subsp. avium subsp. nov., *Mycobacterium avium* subsp. paratuberculosis subsp. nov., and *Mycobacterium avium* subsp. silvaticum subsp. nov. Int. J. Syst. Bacteriol..

[2] Mijs W., de Haas P., Rossau R., Van Der Laan T., Rigouts L., Portaels F., van Soolingen D. (2002). Molecular evidence to support a proposal to reserve the designation *Mycobacterium avium* subsp. avium for bird-type isolates and ‘*M. avium* subsp. hominissuis’ for the human/porcine type of *M. avium*. Int. J. Syst. Evol. Microbiol..

[3] Turenne C.Y., Alexander D.C., Behr M.A., Collins D.M. (2010). *Mycobacterium avium* complex. Paratuberculosis; Organism, Disease, Control.

[4] Inderlied C.B., Kemper C.A., Bermudez L.E. (1993). The *Mycobacterium avium* complex. Clin. Microbiol. Rev..

[5] Lamberski N., Fowler M.E., Miller R.E. (1999). Nontuberculous mycobacteria: Potential for zoonosis. Fowler’s Zoo and Wild Animal Medicine: Current Therapy 4.

[6] Isaza R., Fowler M.E., Miller R.E. (2003). Tuberculosis in all taxa. Fowler’s Zoo and Wild Animal Medicine: Current Therapy 5.

[7] Riggs G., Fowler M.E., Miller R.E. (2012). Avian mycobacterial disease. Fowler’s Zoo and Wild Animal Medicine 7.

[8] Harris N.B., Barletta R.G. (2001). *Mycobacterium avium* subsp. paratuberculosisin veterinary medicine. Clin. Microbiol. Rev..

[9] Whitlock R.H., Buergelt C. (1996). Preclinical and clinical manifestations of paratuberculosis (including pathology). Vet. Clin. North. Am. Food Anim. Pract..

[10] Manning E.J., Sleeman J.M., Fowler M.E., Miller R.E. (2012). Johne’s disease and free-ranging wildlife. Fowler’s Zoo and Wild Animal Medicine: Current Therapy.

[11] Manning E.J., Collins M.T., Fowler M.E., Miller R.E. (1999). Paratuberculosis in zoo animals. Fowler’s Zoo and Wild Animal Medicine: Current Therapy 4.

[12] Hutchings M.R., Stevenson K., Greig A., Davidson R.S., Marion G., Judge J., Behr M.A., Collins D.M. (2010). Infection of non-ruminant wildlife by *Mycobacterium avium* subsp. paratuberculosis. Paratuberculosis; Organism, Disease, Control.

[13] Vansnick E., Vercammen F., Bauwens L., D’Haese E., Nelis H., Geysen D. (2005). A survey for *Mycobacterium avium* subspecies paratuberculosis in the Royal Zoological Society of Antwerp. Vet. J..

[14] Naylor A.D., Richardson D., Sellar M., Harley J., Philbey A.W., Girling S.J. (2018). Clinical Signs, antemortem diagnostics, and pathological findings associated with *Mycobacterium avium* subspecies paratuberculosis infection in Mishmi Takin (*Budorcas taxicolor taxicolor*). J. Zoo Wildl. Med..

[15] Erume J., Spergser J., Rosengarten R. (2001). Rapid detection of *Mycobacterium avium* subsp. paratuberculosis from cattle and zoo animals by nested PCR. Afr. Health Sci..

[16] Witte C.L., Hungerford L.L., Rideout B.A. (2009). Association between *Mycobacterium avium* subsp. paratuberculosis infection among offspring and their dams in nondomestic ruminant species housed in a zoo. J. Vet. Diagn. Investig..

[17] Burgess T.L., Witte C.L., Rideout B.A. (2018). Early-life exposures and Johne’s disease risk in zoo ruminants. J. Vet. Diagn. Investig..

[18] Probst C., Speck S., Hofer H. (2011). Serosurvey of zoo ungulates in central Europe. Int. Zoo Yearb..

[19] Burton M.S., Olsen J.H., Ball R.L., Dumonceaux G.A. (2001). *Mycobacterium avium* subsp. paratuberculosis infection in an addax (*Addax nasomaculatus*). J. Zoo Wildl. Med..

[20] Münster P., Fechner K., Volkel I., von Buchholz A., Czerny C.P. (2013). Distribution of *Mycobacterium avium* ssp. paratuberculosis in a German zoological garden determined by IS900 semi-nested and quantitative real-time PCR. Vet. Microbiol..

[21] Fechner K., Matz-Rensing K., Lampe K., Kaup F.J., Czerny C.P., Schafer J. (2017). Detection of *Mycobacterium avium* subsp. paratuberculosis in non-human primates. J. Med. Primatol..

[22] Fechner K., Schafer J., Munster P., Ternes K., Doring S., Volkel I., Kaup F.J., Czerny C.P. (2017). Detection of *Mycobacterium avium* Subspecies Paratuberculosis in Rock Hyraxes (Procavia Capensis) Imported from South Africa. J. Zoo Wildl. Med..

[23] Zwick L.S., Walsh T.F., Barbiers R., Collins M.T., Kinsel M.J., Murnane R.D. (2002). Paratuberculosis in a mandrill (Papio sphinx). J. Vet. Diagn. Investig..

[24] Bryant B., Blyde D., Eamens G., Whittington R. (2012). *Mycobacterium avium* subspecies paratuberculosis cultured from the feces of a Southern black rhinoceros (*Diceros bicornis minor*) with diarrhea and weight loss. J. Zoo Wildl. Med..

[25] (1998). Proceedings of the Workshop on Diagnosis, Prevention, and Control of Johne’s Disease in Non-Domestic Hoofstock.

[26] Eisenberg T., Wolter W., Lenz M., Schlez K., Zschöck M. (2013). Boot swabs to collect environmental samples from common locations in dairy herds for *Mycobacterium avium* ssp. paratuberculosis (MAP) detection. J. Dairy Res..

[27] Chiodini R.J., Van Kruiningen H.J., Merkal R.S. (1984). Ruminant paratuberculosis (Johne’s disease): The current status and future prospects. Cornell Vet..

[28] Hansen S., Roller M., Alslim L., Böhlken-Fascher S., Fechner K., Czerny C.P., Abd El Wahed A. (2019). Development of rapid extraction method of *Mycobacterium avium* subspecies paratuberculosis dna from bovine stool samples. Diagnostics.

[29] Hansen S., Schafer J., Fechner K., Czerny C.P., Abd El Wahed A. (2016). Development of a recombinase polymerase amplification assay for rapid detection of the *Mycobacterium avium* subsp. paratuberculosis. PLoS ONE.

[30] Rocchetti T.T., Silbert S., Gostnell A., Kubasek C., Widen R. (2016). Validation of a multiplex real-time PCR assay for detection of Mycobacterium spp., Mycobacterium tuberculosis complex, and *Mycobacterium avium* complex directly from clinical samples by use of the BD max open system. J. Clin. Microbiol..

[31] Fechner K., Schafer J., Wiegel C., Ludwig J., Munster P., Sharifi A.R., Wemheuer W., Czerny C.P. (2017). Distribution of *Mycobacterium avium* subsp. paratuberculosis in a Subclinical Naturally Infected German Fleckvieh Bull. Transbound. Emerg. Dis..

[32] Slana I., Kaevska M., Kralik P., Horvathova A., Pavlik I. (2010). Distribution of *Mycobacterium avium* subsp. avium and M. a. hominissuis in artificially infected pigs studied by culture and IS901 and IS1245 quantitative real time PCR. Vet. Microbiol..

[33] Münster P., Völkel I., Wemheuer W., Petschenka J., Wemheuer W., Steinbrunn C., Campe A., Schulz-Schaeffer W.J., Kreienbrock L., Czerny C.P. (2011). Detection of *Mycobacterium avium* ssp. paratuberculosis in ileocaecal lymph nodes collected from elderly slaughter cows using a semi-nested IS900 polymerase chain reaction. Vet. Microbiol..

[34] Münster P., Völkel I., Wemheuer W., Schwarz D., Döring S., Czerny C.P. (2013). A longitudinal study to characterize the distribution patterns of *Mycobacterium avium* ssp. paratuberculosis in Semen, Blood and Faeces of a Naturally Infected Bull by IS 900 Semi-Nested and Quantitative Real-Time PCR. Transbound. Emerg. Dis..

[35] Collins M.T., Oosterhuis J.E. (1993). Diagnosis and control of paratuberculosis in exotic hoofed stock. Proc. Am. Assoc. Zoo Vet..

[36] Münster P., Völkel I., von Buchholz A., Czerny C.P. (2013). Detection of *Mycobacterium avium* subspecies paratuberculosis by is 900-based PCR assays from an alpaca (*Vicugna pacos*) kept in a German Zoological Garden. J. Zoo Wildl. Med..

[37] Matos A.C., Figueira L., Matos M., Pinto M.L., Coelho A.C. (2015). Seroprevalence of *Mycobacterium avium* complex in wild mammals in the Iberian Peninsula. J. Hell. Vet. Med. Soc..

[38] Stevenson K., Alvarez J., Bakker D., Biet F.M., de Juan L., Denham S., Dimareli Z., Dohmann K., Gerlach G.F., Heron I. (2009). Occurrence of *Mycobacterium avium* subspecies paratuberculosis across host species and European countries with evidence for transmission between wildlife and domestic ruminants. BMC Microbiol..

[39] Ball R.L., Kearney C., Burton M.S., Dumoneux G., Olsen J.H. (2002). Morbidity and mortality related to hypoglycemia and chronic energy malnutrition in captive giraffe. Proc. Am. Assoc. Zoo Vet..

[40] Whittington R.J., Begg D.J., de Silva K., Purdie A.C., Dhand N.K., Plain K.M. (2017). Case definition terminology for paratuberculosis (Johne’s disease). BMC Vet. Res..

[41] Whittington R.J., Marsh I.B., Reddacliff L.A. (2005). Survival of *Mycobacterium avium* subsp. paratuberculosis in dam water and sediment. Appl. Environ. Microbiol..

[42] Mondal D., Ghosh P., Khan M.A.A., Hossain F., Böhlken-Fascher S., Matlashewski G., Kroeger A., Olliaro P., El Wahed A.A. (2016). Mobile suitcase laboratory for rapid detection of Leishmania donovani using recombinase polymerase amplification assay. Parasit Vectors.

[43] El Wahed A.A., Weidmann M., Hufert F.T. (2015). Diagnostics-in-a-Suitcase: Development of a portable and rapid assay for the detection of the emerging avian influenza A (H7N9) virus. J. Clin. Virol..

[44] El Wahed A.A., Patel P., Faye O., Thaloengsok S., Heidenreich D., Matangkasombut P., Manopwisedjaroen K., Sakuntabhai A., Sall A.A., Hufert F.T. (2015). Recombinase polymerase amplification assay for rapid diagnostics of dengue infection. PLoS ONE.

[45] El Wahed A.A., El-Deeb A., El-Tholoth M., El Kader H.A., Ahmed A., Hassan S., Hoffmann B., Haas B., Shalaby M.A., Hufert F.T. (2013). A portable reverse transcription recombinase polymerase amplification assay for rapid detection of foot-and-mouth disease virus. PLoS ONE.

[46] Shalaby M.A., El-Deeb A., El-Tholoth M., Hoffmann D., Czerny C.-P., Hufert F.T., Weidmann M., El Wahed A.A. (2016). Recombinase polymerase amplification assay for rapid detection of lumpy skin disease virus. BMC Vet. Res..

[47] Falkinham J.O. (2009). Surrounded by mycobacteria: Nontuberculous mycobacteria in the human environment. J. Appl. Microbiol..

[48] Salchow A. (2018). Untersuchung Zum Vorkommen Und zur Bedeutung Ausgewählter Mykobakterien bei Zootieren. Ph.D. Thesis.

[49] Moser I., Schettler E., Hotzel H., Herzog S., Frölich K. (2011). Mycobacterial infections in free-living cervids in Germany (2002–2006). J. Wildl. D.

[50] Moravkova M., Trcka I., Lamka J., Pavlik I. (2008). A mixed infection of *Mycobacterium avium* subsp. paratuberculosis and M. a. hominissuis in one red deer (*Cervus elaphus*) studied by IS900 BstEII and IS1245 PvuII RFLP analyses: A case report. Vet. Med..

[51] Möbius P., Lentzsch P., Moser I., Naumann L., Martin G., Köhler H. (2006). Comparative macrorestriction and RFLP analysis of *Mycobacterium avium* subsp. avium and *Mycobacterium avium* subsp. hominissuis isolates from man, pig, and cattle. Vet. Microbiol..

[52] Kriz P., Jahn P., Bezdekova B., Blahutkova M., Mrlik V., Slana I., Pavlik I. (2010). *Mycobacterium avium* subsp. hominissuis infection in horses. Emerg. Infect. Dis..

[53] Haist V., Seehusen F., Moser I., Hotzel H., Deschl U., Baumgärtner W., Wohlsein P. (2008). *Mycobacterium avium* subsp. hominissuis infection in 2 pet dogs, Germany. Emerg. Infect. Dis..

[54] Campora L., Corazza M., Zullino C., Ebani V.V., Abramo F. (2011). *Mycobacterium avium* subspecies hominissuis disseminated infection in a Basset Hound dog. J. Vet. Diagn. Investig..

[55] Kim M.-C., Kim J., Kang W., Jang Y.B., Kim Y.-H. (2016). Systemic infection of *Mycobacterium avium* subspecies hominissuis and fungus in a pet dog. J. Vet. Med. Sci..

[56] Hobi S., Bettenay S., Majzoub M., Mueller R., Moser I. (2015). *Mycobacterium avium* subspecies hominissuis infection in a dog from Germany with multifocal alopecia, exfoliative dermatitis, hypercalcaemia and subsequent sebaceous atrophy. Vet. Rec. Case Rep..

[57] Klang A., Staffler C., Mascherbauer C., Spergser J., Rütgen B.C., Hinney B., Luckschander-Zeller N., Kuenzel F. (2014). *Mycobacterium avium* subspecies hominissuis infection in a domestic European shorthair cat. Wien. Tierarztl. Monatsschr..

[58] Shitaye E.J., Grymova V., Grym M., Halouzka R., Horvathova A., Moravkova M., Beran V., Svobodova J., Dvorska-Bartosova L., Pavlik I. (2009). *Mycobacterium avium* subsp. hominissuis infection in a pet parrot. Emerg. Infect. Dis..

[59] Dvorska L., Matlova L., Ayele W.Y., Fischer O.A., Amemori T., Weston R.T., Alvarez J., Beran V., Moravkova M., Pavlik I. (2007). Avian tuberculosis in naturally infected captive water birds of the Ardeideae and Threskiornithidae families studied by serotyping, IS901 RFLP typing, and virulence for poultry. Vet. Microbiol..

[60] Moravkova M., Mrlik V., Parmova I., Kriz P., Pavlik I. (2013). High incidence of *Mycobacterium avium* subspecies hominissuis infection in a zoo population of bongo antelopes (*Tragelaphus eurycerus*). J. Vet. Diagn. Investig..

[61] Wenker C., Wyss F., Hoby S., Ghielmetti G., Friedel U., Gurtner C., Posthaus H. (2018). Non-tuberculous mycobacterial lung infection in an African elephant (*Loxodonta africana*) and a greater one-horned rhinoceros (*Rhinoceros unicornis*) caused by *Mycobacterium avium* ssp. hominissuis and Mycobacterium nebraskense and the reaction to ante- and postmortem tests. Proc. Europ. Assoc. Zoo Vet..

[62] Glawischnig W., Steineck T., Spergser J. (2006). Infections caused by *Mycobacterium avium* subspecies avium, hominissuis, and paratuberculosis in free-ranging red deer (*Cervus elaphus hippelaphus*) in Austria, 2001–2004. J. Wildl. D.

[63] Montali R.J., Mikota S.K., Cheng L.I. (2001). Mycobacterium tuberculosis in zoo and wildlife species. Rev. Sci. Tech..

[64] Cho H.-S., Kim Y.-H., Park N.-Y. (2006). Disseminated mycobacteriosis due to *Mycobacterium avium* in captive Bengal tiger (*Panthera tigris*). J. Vet. Diagn. Investig..

[65] Yong H., Choi G.-E., Lee B.S., Whang J., Shin S.J. (2011). Disseminated infection due to *Mycobacterium avium* subsp. avium in an Asian elephant (*Elephas maximus*). J. Zoo Wildl. Med..

